# Effects of exercise and physical activity on gut microbiota composition and function in older adults: a systematic review

**DOI:** 10.1186/s12877-023-04066-y

**Published:** 2023-06-12

**Authors:** Viviana Aya, Paula Jimenez, Enrique Muñoz, Juan David Ramírez

**Affiliations:** 1 Centro de Investigaciones en Microbiología y Biotecnología de la Universidad del Rosario-UR (CIMBIUR), Facultad de Ciencias Naturales, Universidad del Rosario, Bogotá, Colombia; 2 Facultad de Cultura Física, Deporte y Recreación, Universidad Santo Tomas, Bogotá, Colombia; 3 Molecular Microbiology Laboratory, Department of Pathology, Molecular and Cell-Based Medicine, Icahn School of Medicine at Mount Sinai, New York City, NY USA

**Keywords:** Elderly, Gut microbiota, Gut microbiome, Physical activity, Exercise

## Abstract

**Background:**

The characterization and research around the gut microbiome in older people emphasize microbial populations change considerably by losing the diversity of species. Then, this review aims to determine if there is any effect on the gut microbiota of adults older than 65 that starts an exercise intervention or improves physical activity level. Also, this review describes the changes in composition, diversity, and function of the gut microbiota of older subjects that had improved their physical activity level.

**Methods:**

The type of studies included in this review were studies describing human gut microbiota responses to any exercise stimulus; cross-sectional studies focused on comparing gut microbiota in older adults with different physical activity levels—from athletes to inactive individuals; studies containing older people (women and men), and studies written in English. This review's primary outcomes of interest were gut microbiota abundance and diversity.

**Results:**

Twelve cross-sectional studies and three randomized controlled trials were examined. Independently of the type of study, diversity metrics from Alpha and Beta diversity remained without changes in almost all the studies. Likewise, cross-sectional studies do not reflect significant changes in gut microbiota diversity; no significant differences were detected among diverse groups in the relative abundances of the major phyla or alpha diversity measures. Otherwise, relative abundance analysis showed a significant change in older adults who conducted an exercise program for five weeks or more at the genus level.

**Conclusions:**

Here, we did not identify significant shifts in diversity metrics; only one study reported a significant difference in Alpha diversity from overweight people with higher physical activity levels. The abundance of some bacteria is higher in aged people, after an exercise program, or in comparison with control groups, especially at the genus and species levels. There needs to be more information related to function and metabolic pathways that can be crucial to understand the effect of exercise and physical activity in older adults.

**Trial registration:**

PROSPERO ID: CRD42022331551.

**Supplementary Information:**

The online version contains supplementary material available at 10.1186/s12877-023-04066-y.

## Background

According to the World Health Organization (WHO), the population aged 60 years will double by 2050, and it is projected that people older than 80 will triplicate. These increasing numbers could reach over two billion older adults in the following decades, becoming a significant health issue worldwide to ensure wellness [1].

The aging process is characterized by a progressive loss of physiological integrity, leading to impaired function and increased vulnerability to death [2]. This natural condition affects most living organisms because of the decline of functionality as aging progress, conducted by cellular damage [3]. This deterioration has been widely studied in humans because it is the primary risk factor for significant pathologies [4]. Lopez-Otín and colleagues 2013 enumerated nine candidate hallmarks that represent common aging denominators and contribute to determining the aging phenotype [2]. However, recent studies of aging have planned new hallmarks compromising inflammation and microbiome disturbance, among others [5]. This new perspective could better explain health outcomes related to aging diseases and therapeutic studies to achieve a high-quality lifestyle for older people.

Current advances in sequencing technologies and bioinformatics pipelines have identified notable changes in the gut microbiota/microbiome through the lifespan and its substantial effects on human health [6]. The microbiome refers to the combined genetic material of all the microorganisms (bacteria, fungi, protozoa, megafauna, and viruses) living in a particular environment; this term is explicitly used to denote the genetic and functional diversity of the microorganisms community and its relationship with the host [7, 8].

In brief, the diversity and abundance of taxa that make up the gut microbiota (refers to composition) are highly susceptible to change. This is because of external and internal factors that are inherent to the human being, such as birth mode [9], presence or absence of diseases [10], geographical location [11], and diet [12], among others. Previous research shows that the intestinal microbiota in healthy individuals is stable, especially when there is an absence of clinical manipulation (for example, indiscriminate use of antibiotics) and healthy lifestyle habits, such as an adequate diet and moderate to vigorous physical activity [13]. An adequate balance of bacteria in the digestive tract ensures the microbiota works in a symbiotic environment with the host, however, changes in diversity could lead to a reduction in the abundance of beneficial bacteria and an increase in the prevalence of potentially pathogenic microorganisms, also called dysbiosis [14–17].

Diet is one of the most relevant environmental factors in the investigation of the intestinal microbiome since it modulates the population of microorganisms considerably [18], factors related to diet and nutrition status are key to modulating the composition of microorganisms that inhabit throughout the digestive system [19].

The relationship between the consumption of microbiota-accessible carbohydrates (MACs) and the production of butyrate, as well as the abundance of bacteria that produce this short-chain fatty acid, has been explored in human studies [20]. Significant reductions in the consumption of this macronutrient lead to a drastic decrease in *Bifidobacterium spp*., *Roseburia spp*., and *Eubacterium rectale*. Other microbiota members like *Clostridium spp*. are important for colon cells since they release butyrate as a final product of fermentation. However, the consumption of various starches and fibers can define the type of bacteria that abounds or impacts the intestine [21]. Also, the breaking of large chains of amino acids results in the generation of metabolites such as hydrogen, methane, carbon dioxide, some SCFAs, and branched-chain amino acids (BCAAs). These metabolites resulting from the fermentation of amino acids fulfill a wide range of biological functions for the host; however, the abundance of some of these compounds may be related to inflammation processes or chronic diseases, since large amounts can be detrimental to the intestinal environment [22].

Although it is not clear the underlying mechanisms that drive changes in the gastrointestinal microbiota under exercise conditions, a few studies involving omics sciences provide possible pathways [23–25]. Scheinman et al. identified in a cohort of athletes that the genus *Veillonella* increased considerably after running a marathon. Subsequent analysis of the *V. atypica* strain led the authors to conclude that this microorganism promotes an improvement in race time because of its conversion metabolism of exercise-induced lactate into propionate, thus identifying a natural enzymatic process encoded in the microbiome that enhances athletic performance through the Cori cycle [24]. One of the most relevant results is how intestinal colonization of *Veillonella* increases the Cori cycle by providing an alternative method of lactate processing whereby systemic lactate is converted into SCFAs that re-enter the circulation. SCFAs are absorbed in the sigmoid and rectal region of the colon and enter the circulation through the pelvic plexus, bypassing the liver and draining through the vena cava to reach the systemic circulation directly [24]. Microbiome-derived SCFAs then directly and acutely enhance performance, suggesting that the microbiome might access lactate generated during periods of sustained exercise and convert it into these athletic performance-enhancing SCFAs.

From infancy to old age, the gut microbiome follows some patterns related to rapid change, becoming increasingly unique to individuals as they grow [26]. The characterization and research around the gut microbiome in older adults emphasize microbial populations change considerably by losing the diversity of species [27]; indeed, disturbances and diseases have been linked to these shifts [28, 29].

Recently, three independent cohorts comprising over 9000 individuals aged 18–87 characterized gut microbial patterns associated with age. They performed diversity analysis from multiple samples, paying particular attention to older adults. The results showed amplicon sequence variance (ASV) levels had a unique gut microbiome signature independent of sex or body max index and more related to age [26]. Otherwise, individuals over 80 exhibit continued microbial drift depending on health status. Wilmanski et al., identified microbiome patterns of healthy aging, such as depletion of core genera, primarily *Bacteroides* [26], and different microbial metabolic outputs in the blood, such as lower LDL cholesterol levels, higher levels of vitamin D and beneficial blood metabolites produced by gut microbes. These results are consistent with recent findings showing that host metabolism is crucial to understand the crosstalk between gut communities and the therapeutic alternatives [29, 30]. Despite the diet (a central shifter of the gut community [31, 32]), physical activity status is now considered a relevant factor in the study of the gut microbiome [33].

Physical activity (PA) is any movement produced by skeletal muscles that requires energy expenditure. The WHO includes leisure time, transport to and from places, and workdays as PA [34, 35]. The amount of activity can be quantified between low and vigorous intensity. Some types include walking, cycling, sports, and recreational activities [35], known to prevent and manage chronic non-transmissible diseases (stroke, diabetes, several cancers), many of which appear with aging [36].

PA and exercise training are well-known modifiable factors in aging, either for preventive medicine or chronic disease management. The protective effect and physiological response to exercise training have been extensively described [37, 38]: enhance the antioxidant response, promote activation of anabolic and mitochondrial biogenesis pathways in skeletal muscle [39], decrease inflammatory profile [40], improve insulin sensitivity, myokine profile and endothelial function [41, 42]. These changes confer multiple health outcomes, such as reducing symptoms of anxiety and depression [43], preventing falls and related injuries [41], improving all-cause mortality, an incident of type 2 Diabetes (T2D), specific cancers, or hypertension, and bone and muscular health. Physical exercise is associated with healthy aging, multisystemic benefits provided to this population are condensed in a multidimensional beneficial system; increased muscle synthesis, improved respiratory function, decreased blood pressure levels, and increased neurogenesis, as well as increased bone density muscle mass and loss of body fat percentage [44–46].

Several investigations have repeatedly shown that exposure to regular physical activity confers multiple positive effects on the aging process. The benefits of structured aerobic exercise programs are linked to better learning and cognitive performance on executive function and attentional control in aging [47, 48]. Bouts of physical activity also have a potential therapeutic capacity in conditions related to older adults, such as dementia [49]. Likewise, sufficient results from human and animal trials show the downregulation of pro-inflammatory cytokines and compounds by cardiovascular exercise [47, 50–52]. However, the effect of PA and exercise training on the composition and function of the gut microbiota in older people is not clear, considering the relevant role of the gut commensals for health outcomes and the modifications that confer augmenting PA [53–57].

Cross-sectional [54, 58, 59] and longitudinal [60–62] studies have sought to establish differences in the composition of the human gut microbiota related to physical activity level (PAL); however, the results are highly variable and sometimes contradictory. Only a few results suggest a significant difference in α and β diversity indicators between subjects with high and low PAL [54, 63]; meanwhile, other results show no change in the composition of gut microbiota related to exercise regimen [60, 64]. Modification of single bacteria taxa has been related to exercise stimulus, especially the increased abundance of *Lactobacillus, Bifidobacterium*, and *Akkermansia* [65]. Deeper analysis, specifically metabolome and metagenomic assays, shows significant changes in volatile compounds such as SCFAs [23] and unique members of the microbiome like *Veillonella* [24]*.*

Studies seeking a link between physical activity and the gut microbiota include diverse age groups, such as older people [66–70] young adults [71, 72], adolescents [73], and mostly middle-aged women and men [54, 60, 61, 64, 74, 75]; likewise, diverse frequency, intensity, and type of exercise interventions can be found in these studies [24, 60, 76]. The growing evidence of the modulator effect of physical activity on the gut microbiota makes it relevant to conduct different systematic reviews where the type of population, type of studies, and type of exercise intervention are described.

Therefore, this systematic review aims to identify with the current and evidence whether starting an exercise program or improving PA level brings any notable change in the gut microbiota of adults older than 65 and whether these modifications are reflected in other physiological systems. This systematic review describes the changes in composition, diversity, and function of the gut microbiota of older adults that have improved their physical activity levels.

## Methods

### Criteria for considering studies for this review

#### Types of studies

**Fig. 1:**
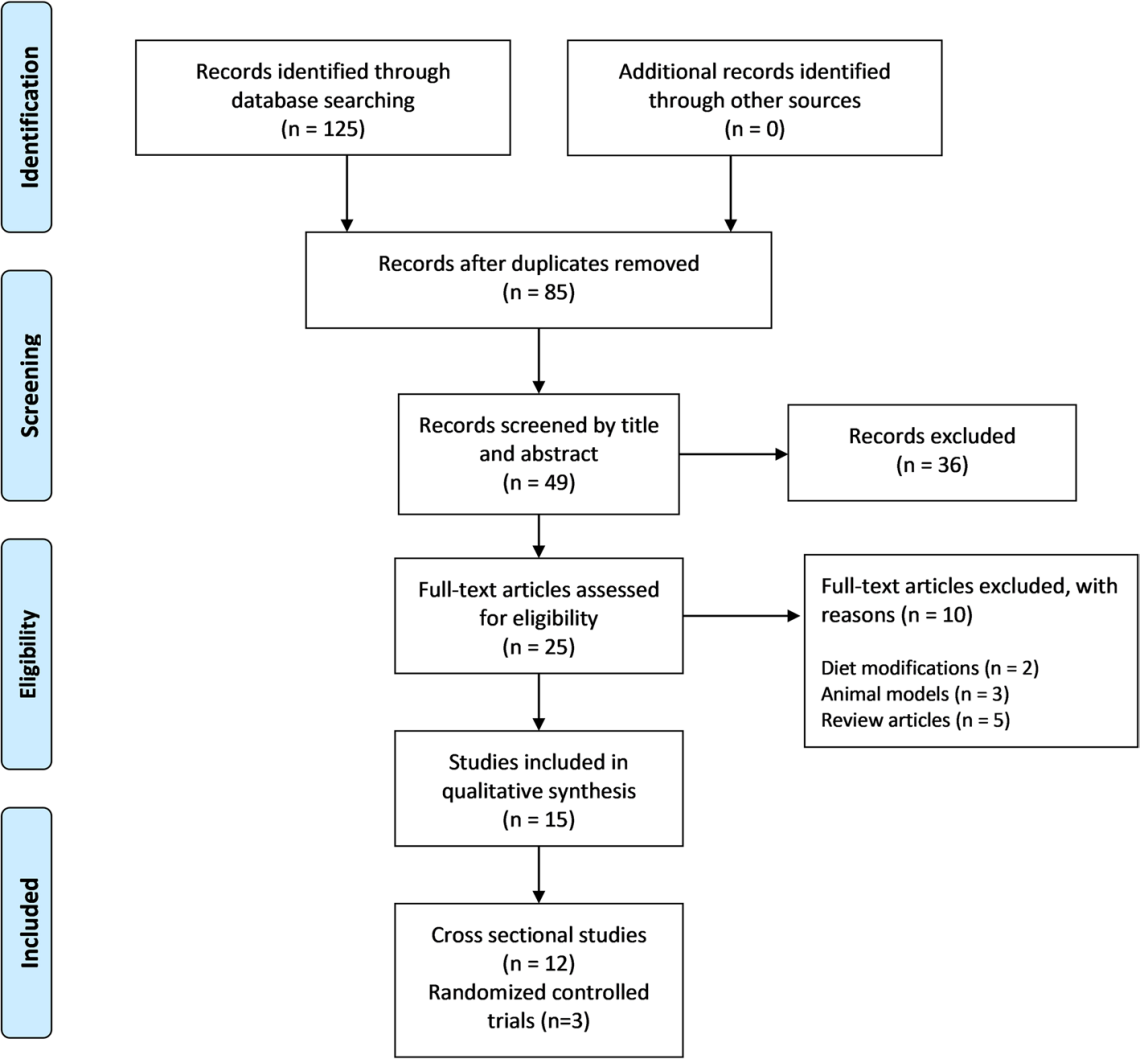
Preferred Reporting Items for Systematic Reviews [78]

Since the gut microbiota research field in physical activity and exercise is growing, past reviews have showed that randomized control studies are few [53, 77]. For that reason, we consider involving: (a) studies describing human gut microbiota responses to any exercise stimulus (b) cross-sectional studies focused on comparing gut microbiota among older adults with different physical activity levels—from athletes to inactive individuals; (c) studies containing older adults women and men (+ 65 years old); (d) studies written in English. We excluded studies containing probiotic or prebiotic consumption and studies focused on diabetes and cancer. Reviews, comments, letters, interviews, and book chapters were also excluded. PRISMA Flow Diagram (Fig. 1) shows the screening process for this systematic review [78].

#### Types of participants

Populations studied in this review were women and men in older adults, which means over 65 years old. Since it is challenging to reach the elderly with no disease or medical condition, we defined our population as aged functional subjects with no physical limitation or physical disability. Studies involving people aged 65 years and older with only two medical conditions related to older adults or healthy were included.

#### Types of interventions

The focus of this review is to determine if starting any exercise intervention could significantly change the gut microbiota; for that purpose, we have established the following eligibility criteria for types of intervention a) randomized controlled trials designed to improve any of the muscular strength, endurance, or flexibility components of fitness in the population named before and b) non-randomized controlled trials designed to improve physical activity level through lifestyle interventions, cross-sectional studies will also be included.

#### Types of outcome measures

The primary outcomes of interest are those related to the diversity and abundance of the gut microbiota. Secondary outcomes will focus on measures or approaches to the function of the gut microbiome. Also, quantification of physical activity level (E.g., median daily step counts) and outcomes related to maximum oxygen consumption and muscular strength will be considered.

The outcomes of interest for this review are:

-Gut Microbiota Abundance: one term frequently used in gut microbiota research is absolute abundance, which refers to the "unobservable actual abundance of a taxon in a unit volume of an ecosystem, such as the gut" [79]. It is essential to highlight that absolute and relative abundance are entirely different terms, according to Lin & Peddada. Changes in the absolute abundance of a single taxon can alter the relative abundance of all taxa [80].

These parameters are determined by the data got in the sequencing process; the next-generation sequencing (NGS) of the 16S rRNA helps describe microbial compositions in a niche. After a quality process, the 16S amplicon sequences can be clustered into Operational Taxonomic Units (OTUs) and Sequence Variants (SVs). In brief, observed counts of OTUs or SVs represent observed abundances of taxa in the sample [79–81].

-Gut Microbiota Diversity: Gut microbiota diversity refers to the number of different species present in a sample, niche, or ecosystem [82]. This review will be focused on stool samples provided by older adults involved in the studies that accomplished the criteria for inclusion. The microbial community in this niche has mainly been characterized in the past years [18]. The bacterial diversity defined by the numerical composition can be calculated with different indexes to determine the changes in the number of species [83]; Alpha diversity refers to the observed richness (number of taxa) and the relative abundances of those taxa (also known as evenness) within a sample. Meanwhile, Beta-diversity is defined as the variability in the microbial community composition among samples [84, 85].

### Search methods for identification of studies

The search strategy is summarized in Table 1. The search terms "Elderly AND Gut Microbiota OR Gut Microbiome AND Physical Activity" were used in the bibliographic databases MEDLINE/Ovid, NIH/PubMed, and Academic Search Complete. This electronic search was done between May 14 and June 15, 2022, and other resources were not identified.

**Table 1:** Search strategy of the systematic review

Database	Search Query
Medline/Ovid	(elderly and (gut microbiota or gut microbiome)).ab. and (physical activity or exercise).ti
NIH/Pubmed	(((elderly [Title/Abstract]) AND (gut microbiota [Title/Abstract])) OR (gut microbiome [Title/Abstract])) AND (physical activity [Title/Abstract])
Academic Search Complete	elderly AND physical activity OR exercise AND gut microbiota

### Quality assessment

Methodological quality and risk of bias for each study were assessed using the Risk Of Bias In Non-randomized Studies—of Interventions tool (ROBINS-I) [86] and the revised tool to assess the risk of bias in randomized trials (RoB 2) [87, 88].

Once a target trial specificity to the study was designed and confounding domains were listed, the risk of bias was explicitly assessed for the comparisons of interest to this review. The overall risk of biased judgment can be found in Supplementary Table 1 and Supplementary Table 2.

## Results

### Description of studies

After the electronic screening and evaluation of the pre-selected studies, we finally included fifteen studies in this review (Fig. 1). The type of study is significant cross-sectional, followed by controlled trials (randomized and non-randomized) and follow-up cohorts that were also included [89]. Table 2 collects relevant information from studies, such as medical conditions, age, and the number of participants who concluded the interventions and/or observations.

**Table 2:** Synopsis of the studies included

Reference	Year	Title	Country	Type of Study	N		Age		Medical condition
					**♀**	**♂**	**Younger**	**Older**	
[49]	2018	Effects of short-term endurance exercise on gut microbiota in elderly men	Japan	Randomized crossover trial		31	62	76	Arterial Hypertension. Dyslipidemia, Hyperglycemia, Prostatic Hyperplasia
[50]	2018	Gut dysbiosis is associated with the reduced exercise capacity of elderly patients with hypertension	China	Cross-sectional	24	32	65	80	Primary Hypertension
[51]	2019	The Association between Objectively Measured Physical Activity and the Gut Microbiome among Older Community-Dwelling Men	United States of America (metropolitan areas)	Cross-sectional		373	78	98	Osteoporotic Fractures
[52]	2019	Aerobic Exercise Training with Brisk Walking Increases Intestinal Bacteroides in Healthy Elderly Women	Japan	12-week non-randomized, comparative trial, where the allocation of the participants to either of the two exercise groups, AE and TM, was based on their preference	29		66	75	Healthy Sedentary Women
[53]	2019	Muscle strength is increased in mice colonized with microbiota from high-functioning older adults	USA	Cross-sectional/Experimental	13	16	70	85	Sedentary older adults, defined as the absence of structured exercise during the previous six months
[54]	2020	Physical fitness in community-dwelling older adults is linked to dietary intake, gut microbiota, and metabolomic signatures	Denmark	Cross-sectional	98	109	65	70	N/A
[55]	2020	Differences in Gut Microbiome Composition between Senior Orienteering Athletes and Community-Dwelling Older Adults	Ireland	Cross-sectional	51	45	68	76	N/A
[56]	2020	Effects of exercise frequency on the gut microbiota in elderly individuals	USA	Data available from American Gut Project (AGP) [57]	897		Normoweight = 462	Overweight = 413	Overweight and Obesity
[54]	2021	The Influence of Different Physical Activity Behaviours on the Gut Microbiota of Older Irish Adults	Ireland	Cross-sectional	100	54	56	69	Cardio Vascular Disease, Type 2 Diabetes Mellitus 7%
[48]	2021	Strenuous Physical Training, Physical Fitness, Body Composition, and *Bacteroides* to *Prevotella* Ratio in the Gut of Elderly Athletes	Slovakia	A cohort of two years (follow-up)	22		63	67	N/A
[58]	2022	Increased physical activity improves gut microbiota composition and reduces short-chain fatty acid concentrations in older adults with insomnia	Israel	Cross-sectional	39	10	LOW 73.66 ± 6.65	HIGH 72.22 ± 5.08	Insomnia
[59]	2020	Effect of an 8-week Exercise Training on Gut Microbiota in Physically Inactive Older Women	China	Randomized controlled trial	6	6	60	75	HbA1c < 6.5%; (3) fasting blood glucose < 7.0 mmol/L; (4) ability to live independently in the community without restrictions on gait or balance; and (5) no diagnosis of type 1 diabetes mellitus or type Two diabetes mellitus
[15]	2021	Effect on the gut microbiota of 1-y lifestyle intervention with the Mediterranean diet compared with energy-reduced Mediterranean diet and physical activity promotion: PREDIMED-Plus Study	Spain	1-year lifestyle intervention	183	179	55	75	HTA
[60]	2022	Effect of Concurrent Training on Body Composition and Gut Microbiota in Postmenopausal Women with Overweight or Obesity	France	Randomized controlled trial	17				N/A
[61]	2022	Exploring the Effects of Six Weeks of Resistance Training on the Fecal Microbiome of Older Adult Males: Secondary Analysis of a Peanut Protein Supplemented Randomized Controlled Trial	USA	Secondary analysis of 14 males that completed six weeks of resistance training		14			N/A

### Results of the search

Likewise, cross-sectional studies did not reflect significant changes in gut microbiota diversity. No significant differences were detected among diverse groups in the relative abundances of the major phyla or alpha diversity measures (Chao 1, Simpson, Shannon; Kruskal–Wallis H test) [70, 89–96].

Otherwise, relative abundance analysis showed a significant change at the genus level in older adults who conducted an exercise program for five weeks or more. The relative abundance of *Clostridioides difficile* was significantly reduced (*P =* 0.03) [97], *Clostridium subcluster XIVa* shows a reduction in women who perform endurance exercise for twelve weeks meanwhile the genus *Bacteroides* shows a significantly increased [98].

When comparing the relative abundance of control and exercise groups, as shown in Table 3, authors inform significant differences in *Bacteroides* and *Subdoligranulum* [89] and a significant increase of *Phascolarctobacterium* and *Mitsuokella* in the exercise group [99]. Differential abundance analysis between two intervention groups conducted at the genus level showed *that Haemophilus, Butyricicoccus, Eubacterium hallii, and Ruminiclostridium* were reduced. In contrast, *Coprobacter*, and uncultured bacterium (from *Rhodospirillales* order) increased in the intervention group compared with the control group (all FDR *P* < 0.1) [28].

**Table 3:** Synthesis of results

Ref	**Type of intervention**		**Type of analysis of the Gut Microbiota**	Diversity metrics		Composition					
	**Exercise Group**	**Control Group**		Alpha diversity	Beta diversity	Relative abundance (Significant differences)					
						Phylum	Class	Order	Family	Genus	Species
[49]	5-week endurance exercise program—with five weeks endurance control group—three cycle ergometer sessions per week	Physical activity level monitoring	16S rDNA amplicon generation Region V3-V4	None of the changes in α-diversity indices were different between the exercise and control periods	The plots indicated that the gut microbial communities were almost identical between the exercise and nonexercise periods (PERMANOVA, *P* > 0.05	NO	NO	NO	NO	↑ *Oscillospira* during the exercise period in the control first group (*P* = 0.003) *↓C. difficile* during the exercise periods in both groups (*P* = 0.03) and (*P* = 0.01)	NO
[50]	Weber’s classification system ^a^: Class A (average exercise capacity), Class B, and Class C (reduced exercise capacity)	Weber classification Class A- B- C	16S rDNA amplicon generation Region V4	No significant differences were detected among the three groups in alpha diversity measures (*p* > 0.05)	Weber A samples were separate from the other groups (ANOSIM pairwise comparisons generated an *R* > 0.5, *p* < 0.05	NO	↑ *Betaproteobacteria* in the Weber A group	↑ *Burkholderiales* and the family *Alcaligenaceae* in the Weber A group	↑ *Ruminococcaceae* in the Weber A group	↑ *Faecalibacterium* in Weber A group ↑ *Escherichia_Shigella* in Weber C group ↑ *Blautia* and *Eubacterium hallii* in Weber B group	↑ *Escherichia coli* in Weber C group
[51]	The objective measure of physical activity with multi-sensor for a typical 7-day period following	N/A	16S rDNA amplicon generation Region V4	No difference in alpha diversity was reported	Step count and self-reported PA were consistently associated with β-diversity as determined by unweighted Unifrac	NO	NO	NO	NO	NO	NO
[59]	Aerobic exercise training (AE) or trunk muscle training (TM)	N/A	Terminal restriction fragment length polymorphism (T-RFLP) analyses	N/A	N/A	NO	NO	NO	NO	↑ *Bacteroides* ↓*Clostridium subcluster XIVa* in the AE group. The relative abundance of C*lostridium cluster IX* was only significantly increased in the TM group	NO
[53]	Short physical performance battery (SPPB)—HF / LF	N/A	16S rDNA amplicon generation—Region V4	Measures of alpha diversity were not significantly different when comparing groups	N/A	NO	NO	NO	↑Prevotellaceae and ↑ Paraprevotellaceae in the HF group	↑*Prevotella*, ↑*Barnsiella*, and ↑*Phascolarctobacterium* in the HF group	*Faecalibacterium prausnitzii, Barnesiella intestinihominis*, *Bacteroides caccae*, and *Clostridium citroniae were* higher in the HF group -*Eubacterium biforme, Desulfovibrio D168*, and *Escherichia coli* were lower in HF
[54]	Two fitness phenotypes, high fitness (HF) and low fitness (LF)	N/A	16S rDNA amplicon generation Region V3	NO	Significant correspondence (*p* = .04) and dissimilarities (*p* = .01) in gut microbiota composition in connection with the two physical phenotypes	NO	NO	NO	NO	NO	NO
[55]	PAL by the FGAS scale: community-dwelling older adults (older adults) and physically active senior orienteers (senior orienteers)	N/A	NGS	No difference in alpha diversity in terms of the Shannon index was observed between the groups	N/A	NO	NO	NO	NO	NO	*Faecalibacterium prausnitzii* and *Bilophila unclassified* were significantly different for 8/15 covariates or combinations of covariates
[56]	E**xercise frequency** Daily exercise group, Regular exercise group (DROE), Occasional exercise group, rare exercise group, never exercise group (NROE)	N/A	Data recover from AGP [57]	OTU numbers were 207.2 and 195.2 (*p* < .001), while the Shannon indices were 5.681 and 5.508 (*p* < .001) in the DROE and NROE groups, respectively. Microbial α-diversity was significantly affected by exercise in overweight individuals	N/A	NO	NO	NO	↓ *Actinomycetaceae, Desulfovibrionaceae, S24-7, Pseudomonadaceae, Barnesiellaceae*, and *Oxalobacteraceae* with exercise frequency. Gradually ↑ *Campylobacteraceae, Fusobacteriaceae, Turicibacteraceae, Paraprevotellaceae, Clostridiaceae, Peptostreptococcaceae, Corynebacteriaceae*, and *Bacteroidaceae* with exercise frequency	NO	NO
[54]	Habitual PA behaviors were assessed by wearing a monitor for 24 h per day	N/A	16S rDNA amplicon generation Regio v3-v4	N/A	N/A	NO	NO	NO	NO	NO	NO
[48]	lifetime high-endurance athletes (LA) and subjects who meet the minimum recommended physical activity levels (CTRL)	N/A	16S rDNA amplicon generation	No difference s in alpha diversity indices between athletes and non-athletes	N/A	NO	↑ Cytophagia (*p* = 0.03) in the CTRL group compared to the LA group	NO	A significantly different abundance of *Bacteroidaceae* in the CTRL group compared to the LA group (*p* = 0.002) and *Clostridiales Incertae Sedis XI* (*p* = 0.01)	Significant differences were observed in the relative abundance of genus *Bacteroides* (*p* = 0.002) between the CTRL and LA group *Phascolarctobacterium* (*p* = 0.01) *Subdoligranulum (p* = 0.02)	*Blautia, Faecalibacterium Bacteroides*, and *Roseburia* formed a significantly different part of all bacteria, 9.3% (7.9–10.8) in the CTRL group compared to 6.2% (5.4–7.2) in the LA group (*p* = 0.001)
[58]	PAL was assessed objectively over two weeks of daily measurement using an accelerometer Step-count: more active/less active groups	N/A	16S rDNA amplicon generation region V4	NO	Microbiota divergence between individuals (Beta diversity, PERMANOVA, *p* < 0.394)	NO	NO	NO	NO	NO	NO
[59]	An 8-week exercise training randomized controlled trial was conducted. This exercise training program consisted of aerobic and resistance exercises	N/A	16S Region V4	NO	N/A	NO	*Clostridia* ↓ compared with the control group after intervention. Significant difference for *Betaproteobacteria* between the two groups (F = 5.149, *P* = 0.047)	*Bifidobacteriales* ↓ of the control group after intervention. *Burkholderiales* showed an increase in the exercise group and a reduction in the control group,	↑*Acidaminococcaceae* in the exercise group, ↑ *Bacteroidaceae* in the control group	*↑***EXERCISE GROUP** *Phascolarctobacterium* and *Mitsuokella* **↓CONTROL GROUP** *Bacteroides* and *Parabacteroides*	
[15]	One year of intervention by promotion of physical activity (IG)	Control group (1-year follow-up	16S rDNA amplicon generation Region V2—V4	NO	N/A	NO	NO	NO	NO	**Intervention Group** ***↓*** *Haemophilus, Butyricicoccus, Eubacterium hallii*, and *Ruminiclostridium 5* **↑***Coprobacter* (FDR *P* < 0.1)	NO
[60]	Training program HIIT + RT: three times per week for 12 wk	CONT	16S rDNA amplicon generation Region V4	NO	PCoA of the unweighted Unifrac distance matrices showed that the pre-and post-intervention microbiota composition changed in most patients from the HIIT + RT group, whereas it remained stable in the CONT group	NO	NO	NO	NO	NO	NO

^a^ Based on the peak VO2 values obtained in test

↑: Increase

↓: Decrease

Similarly, the results described by Magzal et al. [96] in a cross-sectional study including 39 older adults suffering insomnia and classified into the groups high and low PAL shows that *Bifidobacterium*, *Clostridium *sensu stricto* 1*, *Catenibacterium*, *Peptococcus*, *Holdemanella*, and *Butyricicoccus* are among the genera present in more active individuals. Less active people had a higher relative abundance of the genera *Barnesiella*, *Blautia*, *Lachnoclostridium*, *Christensenellaceae R-7* group, and *UCG-005* [96].

Few studies report significant presence or abundance at the species level, Fielding, and collaborators showed that *Faecalibacterium prausnitzii*, *Barnesiella intestinihominis*, *Bacteroides caccae*, and *Clostridium citroniae* were higher in older adults with high fitness profile; meanwhile, a reduction in *Eubacterium biforme*, *Desulfovibrio D168*, and *Escherichia coli* was observed when compared to the Low Fitness group in a cross-sectional study where 29 older women and men (70 years) performed a short physical battery [92].

Various studies aimed to establish a correlation between important indicators of physical activity status such as maximal oxygen consumption or VO2 peak (ml/kg/min) [90, 97, 100], based on the results of cardiorespiratory fitness, older adults were divided into two or three functional groups, where those with higher values oscillate between 22.17 ± 0.51 [90], 23.2 ± 5.8 [100], and 27.3 ± 4.6 (ml/kg/min) [97]. Older people with less VO2 peak values are shown as statistically significantly lower. Correlation analysis exhibit that gut dysbiosis is associated with the reduced exercise capacity of elderly patients with hypertension [90].

Similar findings are observed in a randomized controlled trial [100], where 17 aged adults were assigned to exercise (HIT + RT) or a control group, eventually, after 12 weeks program measurement of VO2max (mL·kg − 1·min − 1) was performed, posterior analysis showed that the Shannon's index was positively correlated with VO2max changes; results suggest an association between microbiota richness and cardiorespiratory fitness improvements [100]. Other correlation analyses between the baseline relative abundance of specific microbiota families and the changes in body composition and cardio metabolic parameters showed that *Bifidobacteriaceae* abundance was positively correlated with fat mass and negatively with muscle mass. Equally, *Paraprevotellaceae* and *Prevotellaceae* were negatively correlated with fat mass and positively with muscle mass [100].

Table 3 shows a trend in the method employed to determine gut microbiota composition using the 16S rDNA amplicon based NGS. Only one selected study indicated Next Generation Sequencing (NGS) [70]. Meanwhile, another reported analyzing available data from The American Gut Project (AGP) [94, 101]. Table 3 summarizes the results related to the diversity and composition of the gut microbiota. Only significant differences between groups are described.

A deeper analysis of functional predictions shows that some metagenomic functions were significantly different between exercise and control periods (*P* < 0.05; FDR < 0.3). Based on the KEGG database, functions related to genetic information processing and nucleotide metabolism were overrepresented after a 5-week endurance exercise program in older Japanese men [97]. A similar analysis shows some crucial differences in 26 metagenomic functions when comparing high-fitness (HF) and low-fitness (LF) aged people. The authors emphasize that the expression of glutathione peroxidase (K00432; GPx) was higher, whereas the remaining 25 functions were lower in HF when compared with LF. GPx was the most highly expressed function (2 to 20-fold increased) compared to all other significant KEGG IDs. [92].

The physical activity frequency is also related to the relative abundance of microbial pathways. Zhu and collaborators suggest that regular exercise significantly modulated microbial function in older people because of the functional analysis performed in samples recovered from the American Gut Project [28]. In synthesis, the relative abundances of 18 pathways were significantly higher. In comparison, the abundances of 5 of those pathways were significantly lower in the daily or regular exercise group (DRE) than in the never or rare exercise group (NRE). These pathways include vitamin-related pathways, nucleotide metabolism-related pathways, glucose metabolism, and amino acid metabolism [94]. Some studies have involved direct quantification methods, such as untargeted metabolomics; Results reported by Castro-Mejía et al., describe significantassociations ( >|0.2| r) for ten gut metabolites and five plasma metabolites with lifestyle co-variables, such as steps per day which correlated positively with mono and di-saccharides metabolism and negatively with amino acid and lipid metabolism. Also, they did not find an essential difference in the concentrations of Short-Chain Fatty Acids (SCFA) from the fecal metabolome according to the high o lower fitness phenotype [93]. In contrast, Magzal, and collaborators report higher concentrations of total SCFA in people with lower physical activity levels [96]; here, acetate was the most prevalent SCFA in both groups. Analysis of the difference in these volatile compounds revealed that the less active group had significantly higher concentrations of propionate, isobutyrate, and valerate compared with the more active group. The magnitude of the difference in concentration between the study groups was higher for propionate (η2 = 16). The less active group also had significantly higher concentrations of total fecal SCFAs, compared to the more active activity group, with a medium effect size (η2 = 08) [96].

Finally, we identified a high variability in the frequency of physical activity both in longitudinal and cross-sectional studies. In brief, randomized trials included exercise protocols between a) 5 weeks of endurance exercise program comprising three ergometer sessions per week [97]; b) Supervised resistance training sessions, twice weekly for six weeks [66]; c) 8-week exercise training randomized controlled trial comprised aerobic and resistance exercise [99]; d) 12-week comparative trials, between aerobic exercise training or trunk muscle training [98]; 12-week training program included high-intensity training and resistance training three times per week [100]. We consider this data is not enough to describe the effect of different exercise intensities and durations on the composition and function of the gut microbiota of older people.

## Discussion

This review summarizes 15 studies involving physical activity, exercise, and gut microbiota changes. In brief, three randomized control trials and 11 cross-sectional trials were analyzed to determine whether performing an exercise program, or higher levels of PA, are consequent to changes in the diversity, abundance, and functional parameters of the gut microbiota of older adults. Similar to reports from other systematic reviews, there are no significant shifts in diversity metrics (Alpha and Beta). Here, only one study from recovery data reported a significant difference in Alpha diversity from overweight people with higher PAL. Contrary to similar findings reported by Barton et al. [63], the microbiota alpha diversity of elderly athletes defined by the Shannon and Simpson index and the Chao1 index did not differ from that of the controls [89].

The abundance of some bacteria is higher in aged people, after an exercise program, or in comparison with control groups, especially at the genus level (Table 3). Some of these bacteria are from the *Lachnospira* and *Lachnospiraceae NK4A136* group, these microbiota members have been described as potentially beneficial [102], because they are producers of SCFA [28], and the synthesis of these organic acids is usually linked to important roles in maintaining colonic host health as an energy source, regulator of gene expression, and anti-inflammatory agents [103], which might be beneficial for the host.

Similar to other studies in non-older adults, some results included in this systematic review suggest that regular exercise significantly modulated microbial function in elderly individuals the data proportionate so far is limited and few studies have included extra analysis such as metabolomic assays or metagenomic approach, where microbial compounds and relative pathways related to physical activity could discover [94], in contrast, other studies including non-older adults have reported significant findings by using specific analysis techniques and combination of omics technologies [69, 104, 105].

Here we highlight the association between the relative abundance of gut microbiota and physical function [99] and a reduced exercise capacity that is negatively associated with the core gut microbiota [90, 91]. We also identified in this systematic review that similar to results presented in cross-sectional studies with young adults [58, 106], consumption of oxygen by older men and women is correlated with species richness and higher diversity of bacterial members of the gut microbiota [90, 97, 100], which reinforce the hypothesis that effect of PAL is more related to functional outcomes rather than compositional indicators (such as diversity or abundance) further investigation is required.

Associations between physical activity and gut microbiota have yet to be extensively studied in older adults. Existing publications focusing on young adults and athletes show consistent results related to the production of SCFAs [63]. Also, bacteria such as *Akkermansia muciniphila* and *Faecalibacterium prausnitzi* have been described in the past [107]. In addition, the health status of older adults involved in biological and gut microbiota studies might be a limiting factor since including people with insomnia [96], Arterial Hypertension, Dyslipidemia, Hyperglycemia, Prostatic Hyperplasia [97], Primary Hypertension [90], Osteoporotic Fractures [91], Overweight and Obesity [94], Cardio Vascular Disease, Type 2 Diabetes mellitus [95] is present in this systematic review. These medical conditions have been reported as modulators of gut microbiota composition [23, 108, 109]. However, data availability for older adults is limited, and comparing healthy and unhealthy subjects could be complicated to perform.

Although the response to exercise and augment of PAL has been extensively studied in diverse biologic systems, such as the mitochondria, the muscle, the liver, and the neurologic system, among others, it is still unclear whether these changes are related to the gut microbiota in older adults. Past reviews and animal studies have linked the possible physiological response to exercise with the community of microorganisms that inhabit the gut [53, 110, 111]. We did not find consistent results that may reflect the modifications of the gut microbiome in other physiological systems. Some bacteria taxa whose abundance changed are beneficial for aged people (Table 3), such is the case of the genus *Oscillospira* which is a promising candidate for the next generation -of probiotics because of its capacity to produce butyrate [112]. *Faecalibacterium* and *Coprococcus* have been correlated with host quality of life indicators in humans diagnosed with depression [113], and some species of the genus *Bacteroides* and *Parabacteroides* are more extraordinary producers of γ-aminobutyric acid (GABA) [114]. Similar findings are described for *Faecalibacterium prausnitzi* that, besides promoting the production of metabolites, have been related to the decrease of inflammatory markers in patients with Alzheimer's-type dementia [115]. *Eubacterium hallii* is also considered a SCFAs producer, especially propionate [116], thanks to metagenomics. It has been discovered that *Subdoligranulum MGS* (metagenomics species) was co-abundantly found with *Akkermansia muciniphila* [117], a promising biomarker for nutritional status [118].

Otherwise, the study provided by Fielding and collaborators [92] looks to describe a correlation between muscle function and gut microbiota through the colonization of mice with microbiota from highly functional older adults. Although results are inconclusive, authors stated that bacteria taxa at the family-level *Prevotellaceae*, genus level *Barnesiella* and *Prevotella*, and species-level *Barnesiella intestine hominis* might be involved in mechanisms related to the maintenance of muscle strength in older adults [92].

**Fig. 2:**
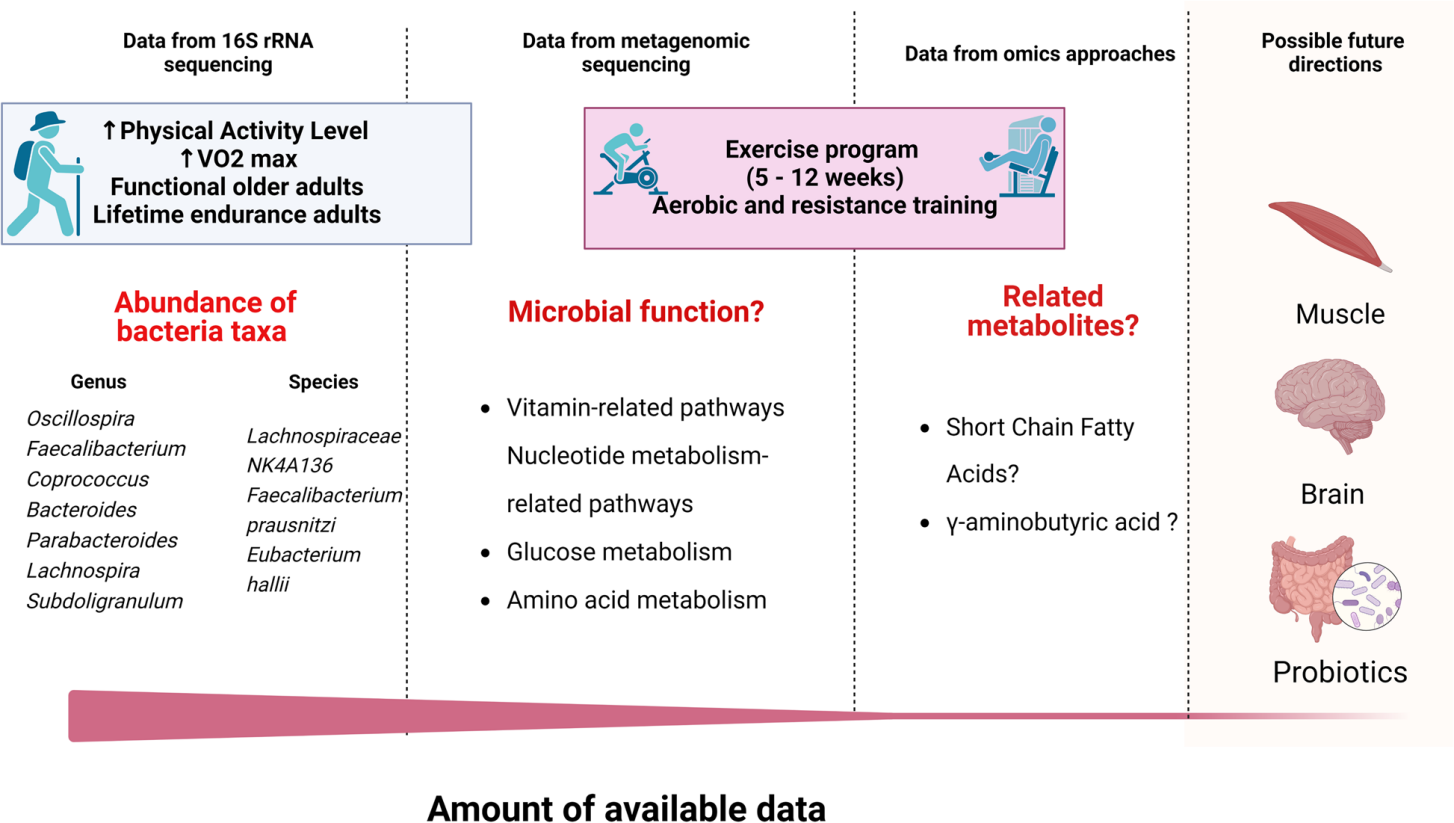
Schematic representation of the data available from studies included in this systematic review. The amount of information available is mostly related to data from 16S rRNA sequencing and the identification of some bacteria associated to beneficial functions for the host; although very few studies used metagenomic approaches, some bacterial functions could be identified in future studies. Here, we identify only two studies describing SCFAs and results are inconclusive. Future directions could link the already known effect of exercise on brain and muscle function in older adults and the gut microbiome

Deeper analysis performed in the studies included identifying metagenomic functions and metabolic pathways to describe some metabolic signatures related to vitamin, amino acid, and glucose functions. In contrast with other reports [33, 119, 120], we did not find an essential association between SCFA and physical activity in older adults. However, very few studies include metabolomic assays, and the data is limited (Fig. 2). This allows us to identify that including diverse sequencing methodologies and the analysis of related metabolites such as SCFAs and GABA in combination with metagenomic approximations could help to describe the role of physical activity on the gut microbiota of older adults in future studies.

Information on taxa and functions related to the benefits of performing PA has been relevant in the identification and isolation of probiotic candidates [121]. Also, including omics techniques, would give insights into the mechanisms underlying the effect of exercise on the gut microbiome of older adults and whether it differs from young people.

## Conclusions

This review aimed to determine if there is any effect on the gut microbiota of adults older than 65 who start an exercise intervention or improve physical activity level. The studies identified do not address this systematic review's objectives. However, almost all the studies analyzed the diversity and abundance of the gut microbiota; there needs to be more information related to function and metabolic pathways that can be crucial to understand the effect of exercise and physical activity in older adults. It is essential to highlight the lack of randomized controlled trials in this field. Most of the studies included are observational, and interventions were mainly voluntary, based on physical exercise (aerobic or muscular) or to increase physical activity through lifestyle changes (increasing the number of steps). The lack of data related to gut microbiota analysis is a weakness that needs to be addressed in future studies.

### Limitations of this review

Authors consider that some limitations of this review included publication bias because of one of the main criteria to report findings related to physical activity and gut microbiota of older adults, which was the statistical significance even though studies with results that do not show statistical significance may be clinically significant, and thus important to the findings of a systematic review. We also consider that the selection and inclusion of cross-sectional studies could be a potential limitation in this review. This is the first time that physical activity, microbiota, and older adults are compared in a systematic review.

## Supplementary Information

**Additional file 1: Supplementary Table 1.** ROBINS-I risk of bias assessment summary: review authors' judgements about each methodological quality item for each non-randomized included study in this review.

**Additional file 2: Supplementary Table 2. **A revised tool to assess risk of bias in randomized trials (RoB 2) summary: review authors' judgements about each methodological quality item for each randomized included study in this review.

## Data Availability

All data generated or analyzed during this study are included in this published article and its supplementary information files.

## Abbreviations

OTUs: Operational Taxonomic Units
WHO: World Health Organization
ASV: Amplicon Sequence Variance
T2D: Type 2 Diabetes
SVs: Sequence Variants
PRISMA: Preferred Reporting Items for Systematic Reviews
AGP: American Gut Project
FGAS: Frändin–Grimby Activity Scale
PAL: Physical Activity Level
NGS: Next-generation sequencing
PA: Physical Activity
HF: High Fitness
LF: Low fitness
HIIT: Hight Interval Intensity Training
RT: Resistance Training
SCFA: Short-Chain Fatty Acids
GABA: γ-Aminobutyric acid
KEGG: Kyoto Encyclopedia of Genes and Genomes
DRE: Regular Exercise Group
NRE: Never or Rare Exercise group
