# Supplementary material for: Effects of exercise and physical activity on gut microbiota composition and function in older adults: a systematic review
Source: BMC Geriatr. 2023 Jun 12;23:364. doi: 10.1186/s12877-023-04066-y (PMC10262510; doi:10.1186/s12877-023-04066-y)
Supplement: Supplementary file 1 — Additional file 1: Supplementary Table 1. ROBINS-I risk of bias assessment summary: review authors' judgements about each methodological quality item for each non-randomized included study in this review. [file 12877_2023_4066_MOESM1_ESM.docx]

**Supplementary Table 1.** ROBINS-I risk of bias assessment summary: review authors' judgements about each methodological quality item for each non-randomized included study in this review.

| **Study** | **Bias due to confounding** | **Bias in selection of participants into the study** | **Bias in classification/**  **measurement of intervention** | **Bias due to deviations from intended interventions** | **Bias because of missing data** | **Bias in measurement of outcomes** | **Bias in selection of the reported result** | **Overall** |
| --- | --- | --- | --- | --- | --- | --- | --- | --- |
| Gut dysbiosis is associated with the reduced exercise capacity of elderly patients with hypertension [59] | High | Moderate | Low | N/A | Moderate | Low | Moderate | Moderate |
| The Association between Objectively Measured Physical Activity and the Gut Microbiome among Older Community Dwelling Men [60] | Low | Moderate | Low | N/A | Low | Low | Moderate | Low |
| Aerobic Exercise Training with Brisk Walking Increases Intestinal Bacteroides in Healthy Elderly Women [61] | Serious | High | Moderate | N/A | High | High | Moderate | High |
| Physical fitness in community-dwelling older adults is linked to dietary intake, gut microbiota, and metabolomic signatures [63] | High | Low | Moderate | N/A | Moderate | Moderate | Moderate | Moderate |
| Differences in Gut Microbiome Composition between Senior Orienteering Athletes and Community-Dwelling Older Adults [64] | Moderate | Low | Low | N/A | Moderate | Low | Moderate | Moderate |
| The Influence of Different Physical Activity Behaviours on the Gut Microbiota of Older Irish Adults [67] | Moderate | Moderate | Low | N/A | Moderate | Moderate | Moderate | Moderate |
| Strenuous Physical Training, Physical Fitness, Body Composition and *Bacteroides* to *Prevotella* Ratio in the Gut of Elderly Athletes [57] | Moderate | Low | Low | N/A | Low | Low | Low | Low |
| Increased physical activity improves gut microbiota composition and reduces short-chain fatty acid concentrations in older adults with insomnia [68] | Low | Moderate | Moderate | N/A | Moderate | Low | Low | Moderate |
| Effect on gut microbiota of a 1-y lifestyle intervention with Mediterranean diet compared with energy-reduced Mediterranean diet and physical activity promotion: PREDIMED-Plus Study [15] | Serious | Moderate | Low | N/A | High | Moderate | Moderate | Moderate |
