# Supplementary material for: Effects of exercise and physical activity on gut microbiota composition and function in older adults: a systematic review
Source: BMC Geriatr. 2023 Jun 12;23:364. doi: 10.1186/s12877-023-04066-y (PMC10262510; doi:10.1186/s12877-023-04066-y)
Supplement: Supplementary file 2 — Additional file 2: Supplementary Table 2. A revised tool to assess risk of bias in randomized trials (RoB 2) summary: review authors' judgements about each methodological quality item for each randomized included study in this review. [file 12877_2023_4066_MOESM2_ESM.docx]

## Supplementary Table 2. A revised tool to assess risk of bias in randomized trials (RoB 2) summary: review authors' judgements about each methodological quality item for each randomized included study in this review
